# Systemic Juvenile Idiopathic Arthritis and Secondary Macrophage Activation Syndrome in Latvia from 2009 to 2020: A Nationwide Retrospective Study

**DOI:** 10.3390/medicina59040798

**Published:** 2023-04-20

**Authors:** Kristīne Lukjanoviča, Ieva Šlēziņa, Zane Dāvidsone, Ruta Šantere, Kristīna Budarina, Valda Staņēviča

**Affiliations:** 1 Department of Pediatric Rheumatology, Children’s Clinical University Hospital, LV-1004 Riga, Latvia; 2 Faculty of Residency, Riga Stradins University, LV-1007 Riga, Latvia; 3 Department of Pediatrics, Riga Stradins University, LV-1004 Riga, Latvia

**Keywords:** juvenile arthritis, systemic juvenile idiopathic arthritis, sJIA, still’s disease, macrophage activation syndrome, MAS, tocilizumab

## Abstract

*Background and Objectives*: Systemic juvenile idiopathic arthritis (sJIA) is a distinctive JIA subtype with mostly nonspecific systemic clinical features, which can be a diagnostic challenge. This study aimed to analyze our experience with sJIA in Latvia for twelve years: assessing clinical and epidemiological characteristics, the efficacy of therapy, and disease outcomes, including the development of macrophage activation syndrome (MAS). *Materials and methods*: This is a descriptive study in which we conducted a retrospective case review of all patients with sJIA diagnosis admitted to the only pediatric tertiary centre in Latvia during the period 2009–2020. *Results:* sJIA was diagnosed in 35 patients with a mean annual incidence rate of 0.85 patients per 100,000 children. Major clinical signs at the first visit were: fever, rash, arthritis, and lymphadenopathy. Almost half of the patients, 48.5%, had a monocyclic disease course, and only 20% of patients had persistent disease. MAS developed in 28.6% of patients. Biological therapy was administered to 48.6% of patients, mostly by tocilizumab, which induced remission in 75% after one year, and in 81.2% after two years without any serious therapy-related complications. In our study, none of the patients had interstitial lung disease, drug reaction with eosinophilia and systemic symptoms (DRESS)-like syndrome, or fatal disease. *Conclusions:* The incidence and clinical characteristics of sJIA correlate with the literature findings, although MAS was more common than described in other studies. There is a tendency for the persistent disease to decrease with the use of biological therapy. Tocilizumab is an efficient choice of treatment with a good safety profile.

## 1. Introduction

Juvenile idiopathic arthritis (JIA) is a term for chronic arthritis of unknown origin, lasting at least 6 weeks with onset before 16 years of age. It is the most common chronic rheumatic condition with variable distribution in different parts of the world, and in some countries, it has not been determined at all; thus, the prevalence of the disease might be much more often than known so far [1,2]. According to the International League of Associations of Rheumatology (ILAR) classification criteria, JIA includes seven categories, defined by the number and location of affected joints, the presence of systemic features and related symptoms, and associated serologies [1,3]. JIA pathogenesis is suspected as a result of genetic predisposition, immune-related factors, and environmental influence; however, specific agents have not been identified [1,2]. 

Systemic juvenile idiopathic arthritis (sJIA) is one of the most common JIA subtypes, accounting for 10–20% of all JIA. It is a distinctive and potentially the most severe form of JIA that differs with pronounced systemic clinical features and is characterized by high spiking fevers, evanescent rash, and frequently chronic polyarthritis. The symptoms are nonspecific and often resemble infection, malignancy, or other inflammatory diseases, making sJIA a diagnostic challenge [1,4].

Although this disease has been described a long time ago by George F Still (1897), the pathogenesis is still unclear. Over the last years, it has become evident that sJIA pathogenesis is driven by the mix of autoimmune and autoinflammatory features, especially driven by IL-1, IL-6, and IL-18, and because of different symptoms, innate system—driven pathogenesis and different treatment efficiency, it is frequently grouped as one of the autoinflammatory syndromes rather than a classical autoimmune disease [4,5,6]. Its disease course can be unpredictable, varying from a monophasic course of the relatively mild disease to chronic relapsing periods of severe polyarthritis accompanied by critical extra-articular symptoms and complications causing significant morbidity and mortality [6].

Macrophage activation syndrome (MAS) is a form of secondary hemophagocytic lymphohistiocytosis—the most devastating complication of sJIA with high fatality rates, so prompt diagnosis and initiation of treatment are of vital importance [1]. MAS is characterized by an overwhelming inflammatory reaction due to an uncontrolled and defective immune response involving T lymphocytes and macrophages, which leads to the overproduction of numerous proinflammatory mediators, thereby eliciting a cytokine storm [6,7]. Clinically, it resembles multiorgan dysfunction and shock and is characterized by non-remitting fever, hepatosplenomegaly, central nervous system involvement, and coagulopathy. Laboratory abnormalities include pancytopenia, hyperferritinemia, elevated serum transaminases, and hypertriglyceridemia [8]. The treatment of MAS is still under debate [6].

The incidence of sJIA and the development of MAS varies widely across the world, but data in Latvia is lacking; thus, no information has been summarized about sJIA and MAS clinical features, disease course, management trends, and outcomes in Latvia until now. This led us to perform a nationwide survey on the sJIA and secondary MAS experience in Latvia.

## 2. Materials and Methods

This is a retrospective, population-based observational study that included patients under 18 years of age who were diagnosed with sJIA (ICD-10 code M08.2) by a pediatric rheumatologist between 1 January 2009 and 31 December 2020. 

The disease was defined as sJIA based mainly on International League of Associations of Rheumatology (ILAR) JIA classification criteria (1995) consisting of the presence of arthritis and fever of at least 2 weeks, plus one of the following criteria: evanescent erythematous rash, generalized lymphadenopathy, enlargement of liver or spleen, or serositis [3]. 

Further, we evaluated whether sJIA patients corresponded to MAS diagnosis according to 2016 EULAR/ACR/PRINTO MAS classification criteria. The identification of MAS can be made in a febrile sJIA patient who has serum ferritin level >684 ng/mL plus any 2 of the following: platelet count ≤181 × 10^9^/L, aspartate aminotransferase >48 units/L, triglyceride concentration >1.76 mmol/L, or fibrinogen ≤3.6 g/L [9]. 

The data were collected from medical records at the Children’s Clinical University Hospital in Riga, which is the sole pediatric hospital in Latvia (a country population of around 1.8 million people); therefore, this study is nationwide. Demographic, clinical, basic laboratory, and treatment data were gathered, and clinical outcomes and complications were observed for at least three years following sJIA diagnosis. Unfortunately, 6 patients did not revisit doctors in our hospital shortly after sJIA onset; therefore, those data are missing. For MAS patients, we collected data regarding clinical signs, possible provoking factors, medication, as well as laboratory markers according to EULAR/ACR MAS criteria 2016 [9]. Data were retrieved by consulting follow-up and hospitalization reports available in the patient’s electronic medical files, and patient checkups were performed at a minimum of every three months. Patients with other chronic diseases that may alter the results and patients with other autoimmune or autoinflammatory syndromes were excluded from this study; none of the study participants had previously received immunosuppressive therapy. The annual incidence for the years 2009–2020 was calculated using the national health statistics database. The data were analyzed using descriptive statistical methods in the Statistical Package for the Social Sciences (SPSS) platform for Windows, Version 26.0. IBM Corp (Armonk, NY, USA). Quantitative data were calculated as a median with an interquartile range, and categorical data were calculated as an absolute number or percentage. An Ethics Committee approval was obtained from the Research Ethics Committee of Riga Stradins University, approval number 22-2/211/2021, dated 26 February 2021.

## 3. Results

A total of 35 patients were diagnosed with sJIA at Children’s Clinical University Hospital between the years 2009–2020. Of those, 57.1% were boys and 42.9% were girls; male to female ratio was 1.3:1. The mean annual incidence was 0.85 (0–2.3) per 100,000 children in Latvia (Table 1).

Autumn was the season with the highest frequency of sJIA onset (42.8%), while spring was the season with the lowest frequency of onset (11.4%); during the summer, the frequency was 25.7%, but in winter months, 20%. 

The median age at diagnosis was 7.3 years (range: 10 months to 17 years). The age distribution was similar in all age groups of sJIA patients: 31% were under 5 years old, 37% were between 5 and 10 years old, and 31% were at least 10 years old. sJIA diagnosis corresponded to ILAR JIA classification criteria (1995) in 75.1% of patients, but all patients (100%) fitted the new provisional classification criteria described in Pediatric Rheumatology International Trials Organization (PRINTO) JIA classification study [10].

The average duration of symptoms prior to diagnosis was 25 days, with a median of 8 days following hospitalization.

The main symptoms during the observation period were those mentioned in the ILAR classification criteria: fever (100%), evanescent rash (91%), arthritis (88%), mostly oligoarthritis, lymphadenopathy (68%), hepatomegaly (62%), and splenomegaly (54%). Clinical features of the study population were determined during the first evaluation of established sJIA diagnosis and at the moment of MAS development and are shown in Table 2.

For most patients (48.6%), the course of the disease was monophasic. We observed the polyphasic course in 22.9% of patients and the persistent disease in 20%, from which only 5.7% had systemic features. For others, only peripheral arthritis persisted. All of our sJIA patients had elevated inflammatory markers: the median erythrocyte sedimentation rate (ESR) was 78 mm/h (Q1, Q3 54 mm/h to 101 mm/h), median C-reactive protein (CRP) was 80 mg/L (Q1, Q3 54 mg/L to 139 mg/L), and median leukocyte count was 18 × 10^9^/L (Q1, Q3 15 × 10^9^/L to 21 × 10^9^/L). Rheumatoid factor was negative in all cases, but antinuclear antibodies were positive in 34.3% of patients.

The development of MAS was observed in 10 (28.6%) sJIA patients, and in all cases, it was presented during the first sJIA acute episode, in two cases as the primary diagnosis before sJIA was established. It was diagnosed a median of 7 days after clinical and laboratory signs of deterioration appeared in sJIA patients. Bone marrow biopsy was performed for eight (80%) of MAS patients, and our pathologists confirmed hemophagocytosis signs in two (25%) bone marrow specimens. Most MAS patients did not receive any immunosuppressive therapy before; 3/10 were already on corticosteroids. During the development of MAS, some patients had a concomitant episode of acute infection, of whom two patients had gastroenteritis (Rotavirus, Norovirus), two patients had Herpes simplex virus (Herpes labialis) clinically, other two patients had upper respiratory tract infection, and one patient had pneumonia (undetermined etiology). There is no direct evidence that infection was involved in the development of MAS. One patient had a spleen rupture before symptoms appeared, but no other diseases were associated with MAS.

We collected data on laboratory changes and clinical signs when the MAS diagnosis was made, and those are reported in Table 2 and Table 3. The median ferritin level was 11,551 ng/mL.

All of the sJIA patients were treated with combined therapy (Table 4), which initially included corticosteroids (CS) or/and non-steroid anti-inflammatory drugs (NSAID). Some sJIA patients achieved remission with CS and methotrexate only. For those who had a recurrent, persistent disease or MAS developed, biological therapy was added, mostly tocilizumab (in 45.7% of patients). 

All MAS patients (100%) received methylprednisolone pulse therapy together with cyclosporine, on which 20% of patients achieved complete remission of the disease. For the other 80% of patients, tocilizumab was added to therapy, and in 20% of patients, MTX was given as there was no or only partial improvement with initial therapy. In all those who used tocilizumab, the disease flare was stopped a couple of days after initiation of medication.

The interval of time in which the medication was discontinued is summarized in Table 5. Corticosteroid use in most patients (19/29, 65%) was stopped less than a year after the onset of the disease, and cyclosporine was stopped during the first two years in all patients (10/10, 100%). Longer-term use was mostly observed in the methotrexate group (9/22, 40%) and biological treatment group (4/17, 23%), where it was used for at least three years.

Data on patients’ remission and complications were collected three years after disease onset. Of those who were under supervision, the majority (82.8%) achieved remission in the first year of the disease, mostly with medication, and only 6.9% of patients were under remission without medication. Three years after disease onset, most children (75.9%) were in remission, 55.2% without, and 20.7% with medication.

Tocilizumab was the most frequently used biological therapy in cases of persistent/recurrent disease or MAS. All patients who received tocilizumab were under our supervision for at least 3 years. Of those who used tocilizumab, remission after one year was achieved in 75%, and 81.2% after two years, of which 18.7% were in remission without medication. 

In the case of sJIA-associated MAS, remission after 1 year was achieved in 80% of patients and 90% after 2 years.

Complications were mostly associated with corticosteroid use: exogenous Cushing syndrome developed in 55.1%, bacterial infections developed in 6.9%, and one case of prolonged QT interval in electrocardiogram after initiation of methylprednisolone pulse therapy was observed. Some other complications, such as rash, growth delay, delayed puberty, and glaucoma, were also observed. There was 1 case of cyclosporine-induced seizures, which stopped after discontinuation of the medication.

No serious complications were associated with the use of tocilizumab; however, there was one case of possible acne development after initiation of tocilizumab and one case with possible non-severe secondary thrombocytopenia.

Our study showed no cases of JIA-associated uveitis, drug reaction with eosinophilia and systemic symptoms (DRESS)-like syndrome, lung disease, or fatalities.

## 4. Discussion

This is the first study of sJIA and secondary MAS in Latvia. The mean annual incidence that was established in our study was similar to that described in other European countries. In our study, it was 0.0–2.3 sJIA cases per 100,000 children in Latvia per year (average 0.85/100,000). Recently in Germany, it was 2/100,000, in Estonia 0.9/100,000, and in Catalonia 0.5/100,000, but in earlier studies in Europe, it was 0.4–0.9 per 100,000. The incidence of sJIA appears to be constant over the past 20 years [11,12,13,14].

In 2019, PRINTO proposed a new classification criteria study for JIA as the revision of ILAR 1995 criteria and is now undergoing a validation process [10]. In the sJIA case, less strict criteria were proposed to allow earlier diagnosis and treatment. In these criteria, fever might be reoccurring during a 2-week timeframe, thus present on some days and absent on other days. Additionally, arthritis might last less than 6 weeks or might be absent at all [10,15]. In our study, we recognized that all our patients (100%) corresponded to new provisional criteria, but only 75.1% to ILAR 1995 JIA criteria, mostly due to the absence of arthritis. We, therefore, suspect that the new provisional criteria may be more sensitive.

The main presenting symptoms in our study, namely fever, rash, arthritis, and hepatosplenomegaly, were similar to those reported in studies conducted abroad and haven’t changed significantly from those described by Still in 1897 [14,16].

As sJIA occasionally progresses to life-threatening complication MAS, timely diagnosis is crucial. The mortality rate of MAS in the case of sJIA is 8–17% [17]. In our study, 28.6% of patients developed MAS, a higher percentage than described in other studies (around 7–13% clinically, up to 40% subclinically) [18,19]. This may be due to increased recognition of MAS nowadays, as earlier studies describing MAS epidemiology were carried out prior to the development of EULAR/ACR/PRINTO 2016 classification criteria for MAS complicating sJIA [18,19,20]. Another explanation may be that in our study, biological treatment was not applied early on in the course of the disease. Although we observed MAS more often, no fatalities or refractory MAS were observed in our study, which could be due to the relatively fast determination of MAS diagnosis (median seven days after clinical/laboratory signs appeared). It should be noted that distinguishing a genuine MAS from a “simple” sJIA flare remains challenging, despite the existing MAS criteria, and is a major management issue [7].

In our study, all patients with MAS initially received the combination of therapy with methylprednisolone in pulse dosage and cyclosporine, but for 80% of patients, tocilizumab was added with a full remission regarding the flare of the MAS and systemic symptoms. Standardized diagnostic treatment guidelines for MAS in sJIA are currently lacking, and treatment of MAS in sJIA relies more on experience than evidence-based medicine [6]. Most patients described in the literature have received methylprednisolone pulse dose, cyclosporine, etoposide, or from biological therapy, mostly anakinra, with a good outcome [6,21]. The efficacy of tocilizumab in the case of MAS is scarce [22].

Before the era of biological treatment (Il-1 and Il-6 inhibitors), a large proportion of sJIA patients developed severe chronic disease with persistent inflammation and erosive polyarthritis. As new biologic medications have been available for the last two decades in Latvia, there has been a decrease in the persistent disease course. Similar to foreign studies, the persistent disease is observed in around 20% of sJIA patients, which is a significant decrease if compared with the pre-biological era, when it was around 50–80% [23,24].

In our study, of biological therapy we mainly used was Il-6 receptor inhibitor tocilizumab, as it was one of the first available biological medications in Latvia. The efficacy was similar to other studies abroad and comparable to anakinra, which is the first choice in some guidelines [25]. No cases of serious side effects were observed, and tocilizumab was generally well tolerated. According to our disease outcomes and the tocilizumab safety profile, the use of tocilizumab is supported. However, in the last few years, pediatric rheumatologists have hypothesized that using recombinant Il-1 receptor antagonist (anakinra) as first-line therapy in new-onset sJIA patients is a highly efficacious strategy to induce and sustain inactive disease and prevent disease and corticosteroid related damage in sJIA [26]. As in our study, all patients took corticosteroids, and some of them had serious corticosteroid-related complications; early onset treatment with anakinra could be a new strategy for treating patients with sJIA in our hospital. Another alternative for the treatment of sJIA is the Il-1β monoclonal antibody canakinumab, which, in some studies, is mentioned as the most effective treatment option [27]. Currently, this medication is not available in Latvia, which we expect will change in the future. 

For the last 15 years, pediatric rheumatologists have increasingly detected cases of specific lung disease (mostly interstitial lung disease (ILD), pulmonary arterial hypertension, or pulmonary alveolar proteinosis) associated with sJIA, which was rarely seen before. This new entity is associated with DRESS-like syndrome and severe reaction to biological treatment, and it has an alarmingly low survival rate (5-year survival rate of around 42%) [28]. The etiology of these entities has not been clarified, but theories of their association with biological therapy exposure (Il-1 and Il-6 inhibitors) are discussed and under research [28,29]. In our study, we did not observe any cases of lung disease, severe reaction to biological treatment, or DRESS-like syndrome.

### 4.1. Limitations

This study possesses some limitations of note. First, the retrospective approach of this study limits the data available to what was registered in the medical records; thus, some data might be missing, or some manifestations and diagnostic tests of interest were not performed, for example, bone marrow biopsy. Second, the small research population in this study is a statistical drawback, although, for such a small population, this number of patients is significant. Third, diagnostic approaches and treatment practices vary among physicians, which may be affected by a certain degree of subjectivity and thus have had effects on diagnostic results, disease response, and course. However, all the patients in this cohort were seen by an experienced pediatric rheumatologist with knowledge of sJIA and MAS. Fourth, the follow-up period of 3 years was not long enough to determine the outcome of all patients with sJIA and secondary MAS. Fifth, the patient data were collected over the course of 13 years, and the knowledge of sJIA and MAS, as well as treatment strategies, have changed and improved over time. Finally, and the latest, our study includes data from only one center, but it should be noted that sJIA and MAS in Latvia are treated in the only tertiary children’s hospital; therefore, data are representative of the Latvian population.

### 4.2. Future Directions

Our future direction would be to do a prospective trial with our sJIA patients to track long-term disease outcomes, especially for patients with persistent/recurrent disease, and to observe the efficacy and side effects of different biological therapy options in the distant future, also after the transition to adult care.

An important plan in the future would be to create an electronic national registry/database for pediatric rheumatology patients in Latvia, including data about JIA patients, with the aim to improve patient care and facilitate research in epidemiological and other types of studies in the future.

## 5. Conclusions

This is the first epidemiological study of sJIA and secondary MAS in Latvia. The incidence rates of sJIA are similar to those reported for other European countries. Complications with MAS were seen more frequently, but mortality was not observed at all, unlike those described in other studies. Tocilizumab was the most commonly used biological medication with a good result in achieving remission of the disease and a notable tendency for the persistent sJIA to decrease. The DRESS-like syndrome and lung disease were not observed in our study as it is elsewhere.

## Figures and Tables

**Table 1:** The mean annual incidence of sJIA in Latvia.

Year	No. of Patients	Children in Latvia	Incidence Per 100,000 Children
2009	3	388,449	0.8
2010	0	353,357	0
2011	4	360,216	1.1
2012	3	332,979	0.9
2013	4	347,018	1.2
2014	3	325,425	0.9
2015	8	348,660	2.3
2016	3	330,414	0.9
2017	0	356,527	0
2018	4	338,102	1.2
2019	2	358,813	0.6
2020	1	340,806	0.3

**Table 2:** Clinical features of sJIA patients and patients during MAS episodes.

sJIA		MAS
**Fever**	100% (35/35)	100% (10/10)
Evanescent rash	91.4% (32/35)	90% (9/10)
Arthritis/arthralgia	88.6% (31/35)	80% (8/10)
Oligoarthritis	45.7% (16/35)	30% (3/10)
Polyarthritis	34.3% (12/35)	30% (3/10)
Arthralgia	8.6% (3/35)	20% (2/10)
Lymphadenopathy	68.6% (24/35)	100% (10/10)
Hepatomegaly	62.9% (22/35)	50% (5/10)
Splenomegaly	54.3% (19/35)	50% (5/10)
Myalgia	31.4% (11/35)	50% (5/10)
Serositis	14.3% (5/35)	50% (5/10)
Hemorrhagic rash	17.1% (6/35)	30% (3/10)
Respiratory involvement	11.4% (4/35)	-
Gastrointestinal involvement	8.6% (3/35)	-
Cardiac involvement	8.6% (3/35)	-
Eye involvement	8.6% (3/35)	10% (1/10)
Urogenital involvement	2.9% (1/35)	-
Nervous system involvement	2.9% (1/35)	20% (2/10)

**Table 3:** Laboratory results in sJIA patients during MAS episode.

MAS Laboratory Criteria	Ferritin	Thrombocytes	ASAT	Triglycerides	Fibrinogen
	>684 ng/mL	≤181 × 10^3^/L	>48 U/L	>1.76 mmol/L	≤3.6 g/L
Patient 1	20,428	46	268	3.27	0.56
Patient 2	1479	1174	480	N/A	2.57
Patient 3	2722	231	42	2.52	3.08
Patient 4	14,611	98	231	4.92	0.96
Patient 5	5246	222	560	2.24	0.76
Patient 6	14,097	142	904	2.77	1.3
Patient 7	18,278	42	89	2.86	0.58
Patient 8	13,246	232	27	1.02	4.04
Patient 9	19,477	102	127	2.2	3.7
Patient 10	9856	175	80	2.18	2.59

**Table 4:** Pharmacological treatment in sJIA patients and patients during MAS episode.

sJIA		MAS
NSAID ^1^	94.3% (33/35)	90% (9/10)
Corticosteroids	100% (35/35)	100% (10/10)
Pulse form	94.3% (33/35)	100% (10/10)
Peroral form	100% (35/35)	100% (10/10)
Methotrexate	74.3% (26/35)	20% (2/10)
Biological therapy	48.6% (17/35)	80% (8/10)
Tocilizumab	45.7% (16/35)	80% (8/10)
Anakinra	2.9% (1/35)	-
Cyclosporine	28.6% (10/35)	100% (10/10)

^1^ Non-steroid anti-inflammatory drugs.

**Table 5:** The interval of time when specific medication was stopped.

Time Interval in Years	The Patient Number Who Stopped Medication in the Specified Time Interval			
	Methotrexate	Corticosteroid	Cyclosporine	Biological Therapy
<1y	5	19	8	2
1-<2y	4	7	2	9
2-<3y	4	2	0	2
≥3y	9	1	0	4

## Data Availability

The data that were presented in this study are available on request from the corresponding author. The data are not publicly available due to privacy restrictions.
